# Quality of Reporting of Bioequivalence Trials Comparing Generic to Brand Name Drugs: A Methodological Systematic Review

**DOI:** 10.1371/journal.pone.0023611

**Published:** 2011-08-17

**Authors:** Amélie van der Meersch, Agnès Dechartres, Philippe Ravaud

**Affiliations:** 1 INSERM, U738, Paris, France; 2 AP-HP (Assistance Publique des Hôpitaux de Paris), Hôpital Hôtel Dieu, Centre d′Épidémiologie Clinique, Paris, France; 3 Université Paris Descartes, Sorbonne Paris Cité, Faculté de Médecine, Paris, France; Children's Hospital of Eastern Ontario, Canada

## Abstract

**Background:**

Generic drugs are used by millions of patients for economic reasons, so their evaluation must be highly transparent.

**Objective:**

To assess the quality of reporting of bioequivalence trials comparing generic to brand-name drugs.

**Methodology/Principal Findings:**

PubMed was searched for reports of bioequivalence trials comparing generic to brand-name drugs between January 2005 and December 2008. Articles were included if the aim of the study was to assess the bioequivalency of generic and brand-name drugs. We excluded case studies, pharmaco-economic evaluations, and validation dosage assays of drugs. We evaluated whether important information about funding, methodology, location of trials, and participants were reported. We also assessed whether the criteria required by the Food and Drug Administration (FDA) and the European Medicine Agency (EMA) to conclude bioequivalence were reported and that the conclusions were in agreement with the results. We identified 134 potentially relevant articles but eliminated 55 because the brand-name or generic drug status of the reference drug was unknown. Thus, we evaluated 79 articles. The funding source and location of the trial were reported in 41% and 56% of articles, respectively. The type of statistical analysis was reported in 94% of articles, but the methods to generate the randomization sequence and to conceal allocation were reported in only 15% and 5%, respectively. In total, 65 articles of single-dose trials (89%) concluded bioequivalence. Of these, 20 (31%) did not report the 3 criteria within the limits required by the FDA and 11 (17%) did not report the 2 criteria within the limits required by the EMA.

**Conclusions/Significance:**

Important information to judge the validity and relevance of results are frequently missing in published reports of trials assessing generic drugs. The quality of reporting of such trials is in need of improvement.

## Introduction

To lower healthcare costs, substituting brand-name drugs for low-cost bioequivalent generic drugs has greatly increased. In the United States, generic drugs represented 47% of all prescriptions dispensed in 1999, 61% in 2006 and 69% by the end of 2008 [1]. According to the Generic Pharmaceutical Association, the use of generics saved the US healthcare system approximately $121 billion in 2008. Generic and brand-name drugs have the same active principle, but generic drugs could differ in inert binder, color of tablet and manufacturing process. To approve new generic drugs, the US Food and Drug Administration (FDA) and the European Medicine Agency (EMA) relies on the results of bioequivalence trials based on pharmacokinetic criteria: rate of absorption as determined by the peak plasma concentration (C_max_), area under the plasma concentration–time curve from time 0 to time t = end of the study (AUC|_0 to t_) and to infinity (AUC|_0 to infinity_). The FDA requires the 90% confidence intervals (CIs) of the ratio of the generic and brand name drugs for these 3 criteria to be within 80% and 125% [2] whereas the EMA [3] requires only Cmax and AUC|0 to t. Reports of some studies and letters have warned about generic-drug substitution, suggesting lower efficacy and higher rates of adverse events [4], [5], [6], [7], [8], [9], but data are scarce, and most studies had a low level of evidence. More importantly, drug companies may be implicated in this anti-generic-drug campaign [10]. Recently, 2 meta-analyses [11], [12] comparing the efficacy of brand-name and generic drugs in the field of cardiovascular diseases and seizures did not show any superiority of the brand-name over the generic drugs.

In this study, we aimed to investigate published reports of bioequivalence trials comparing generic to brand-name drugs to assess their quality of reporting.

## Methods

### Search strategy

We performed an electronic search of MEDLINE via PubMed to identify all reports of trials published from Jan. 1, 2005, to Dec. 31, 2008. The following search equation was used: "Therapeutic Equivalency"[Mesh] OR "Drugs, Generic"[Mesh] OR "Biological Availability"[Mesh] OR "Intellectual Property"[Mesh] OR non proprietary OR brand name OR innovator AND (Humans[Mesh] AND English[lang] AND ("2005/01/01"[PDat]: "2008/12/31"[PDat])). We also electronically searched the table of contents of journals (Arzneimittel-forschung, International Journal of Clinical Pharmacology and Therapeutics, Clinical Therapeutics) known to publish reports of bioequivalence trials assessing generic drugs for additional references missed during the PubMed search.

### Selection of relevant articles

One of us (A.V.D.M) selected potentially relevant articles after screening the titles and abstracts. In case of uncertain eligibility, the full text was screened. Articles were included if the aim of the study was to assess the bioequivalency of generic and brand-name drugs. We excluded case studies, pharmaco-economic evaluations, meta-analyses, commentaries and validation dosage assays of drugs. Studies were also excluded if the active principle was not approved by the FDA or EMA and if they involved animals or *in vitro* analysis.

If information about the products being tested lacked, FDA or EMA websites were searched to obtain further information.

A second reviewer (A.D) assessed all articles of uncertain eligibility, and the final decision for inclusion was obtained by consensus between the 2 reviewers.

### Data extraction

We generated a standardized data collection form based on the guidelines of the FDA [2] and EMA [3], a review of the literature and *a priori* discussion. Before data extraction, as a calibration exercise, 2 members of the team (A.D., A.V.D.M) evaluated a random set of 10 reports. All disagreements were resolved by consensus, and the form was modified accordingly.

The following data were extracted from each article:

1. General characteristics of the selected studies: category of the journal (pharmacology, general medicine, specialty medicine) and year of publication; whether the funding source was reported and whether it was public or private.
2. Characteristics of the drugs: the class of drug according to the Anatomical Therapeutic Chemical classification [13]; the name of the reference drug and whether the drug had a narrow therapeutic index (NTI) (i.e., a drug with less than a 50% difference between the minimum toxic concentration, minimum effective concentration in blood and median effective dose[14]) and whether this information was reported in the article. For NTI drugs, the EMA requires narrower limits than for non-NTI drugs: the 90% CIs for the generic to brand-name ratio should be between 90% and 111% instead of between 80% and 125%.[3].
3. Study design: whether the study design was reported, the type of design (i.e., randomized controlled, parallel or cross-over), and the sample size. We also noted whether a registration number in an international database was reported.
4. Administration of the drugs: whether the mode of administration of the drugs was reported: the route of administration (i.e., oral, parenteral), the dose of the drugs (i.e., single or multiple) and how the drugs were administered (i.e., under fasting or fed condition).
5. Methodology of the study: whether the methods used to generate the randomization sequence and conceal allocation were reported and adequate according to the definitions provided by the Cochrane Collaboration [15]; whether study participants, care providers and outcome assessors were reported to be blinded to the treatment arm; whether the number of participants excluded from data analysis and the reasons for exclusion were reported; whether a sample size calculation was reported; and whether the statistical analysis performed was reported and the type of analysis (i.e., ANOVA, *t* test).
6. Setting and population characteristics: whether the article reported the country where the study was performed and the main characteristics of the study participants (age, weight, height, sex, exclusion criteria).
7. Results and conclusions: the results of the trial (i.e., 90% CI of the generic to brand-name ratio for C_max_, AUC|_0 to t_ and, AUC|0 _to infinity_ of the generic to brand-name drug and the conclusions (i.e., bioequivalence, non-bioequivalence, unclear).

The same reviewer (A.V.D.M) independently completed all the data extractions. A second member of the team (A.D.) reviewed a random sample of 30 articles to assess inter-rater reliability.

PRISMA checklist is provided in Appendix S4.

### Statistical analysis

The analysis was descriptive. Data were summarized as number and percentages for qualitative variables, and median, interquartile range (IQR) and range for continuous variables. Inter-rater agreement between the 2 assessors (A.V.D.M. and A.D.) was assessed by the kappa coefficient for qualitative variables. All analyses involved use of R v2.9.0.

## Results

### Selection of relevant trials

**Figure 1:**
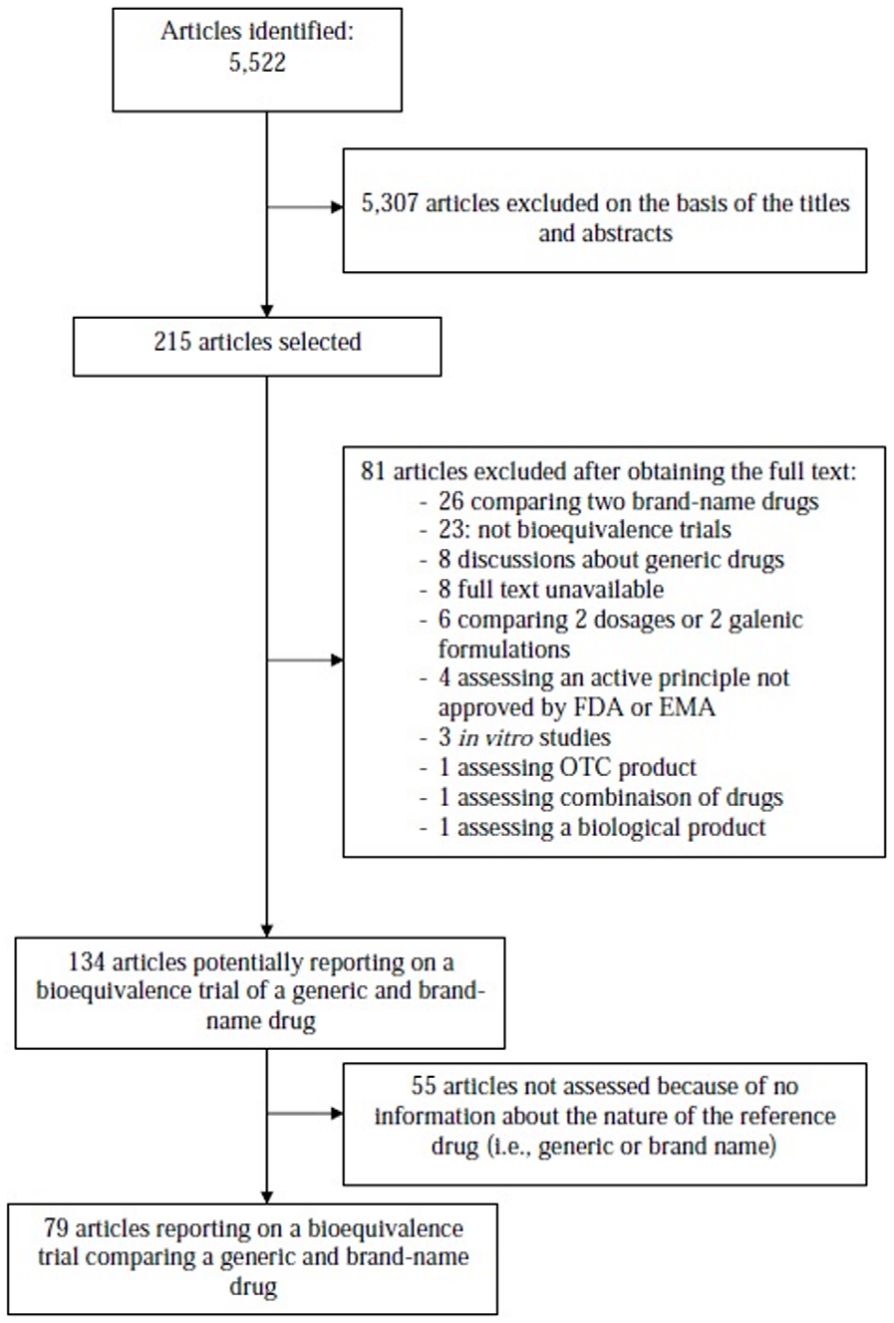
Flow diagram of the selection of reports of trials comparing generic to brand-name drugs published between 2005 and 2008.

A flow chart of the selected articles of trials comparing generic to brand-name drugs is in Figure 1. Briefly, the electronic search yielded 5,522 citations, 215 articles were selected for further evaluation, and a final 79 reports of bioequivalence trials were selected after reading the full text. Of these, 63 articles described non-NTI drugs (Appendix S1) and 16 NTI drugs (Appendix S2). Only 5 articles reported that the drug was an NTI drug. In total, 55 articles (Appendix S3) out of 134 (41%) were eliminated because only the compendium name of the reference drug was reported, and we could not determine whether the reference drug was a brand-name or another generic drug. In general, the kappa for data extraction was good, ranging from 0.71 to 1.

### General characteristics of the trials

Table S1 provides the general characteristics of the selected bioequivalence trials. Most of the studies were published in pharmacology journals (78%). The studies concerned a wide range of pharmacological areas, the most common being study of anti-infective agents for systemic use (37%) and of the cardiovascular system (18%) and nervous system (14%).

Funding was reported in 41% of the articles and was private in 25%. In total, 73 reports (92%) described single-dose administration and 6 (8%) multiple-dose administration. In 66 (83%), the administration was under the fasting condition.

### Study design and methodology

Table S2 shows the study design and methodology of the bioequivalence trials. Only one trial reported a registration number in an international database. The study design was reported in most articles (99%) and was a randomized cross-over controlled trial in these studies. The median sample size was 24 (IQR 24 to 28; range 12 to 70). A sample size calculation was reported in 27 articles (34%). In one article, the authors reported that they had increased the number of participants during the study because they could not conclude bioequivalence with the initial sample size.

The method used to generate the randomization sequence was reported and adequate in 12 articles (15%). Allocation concealment was reported and adequate in 4 articles (5%). Blinding of study participants, care providers and outcome assessors was reported in 13 (16%), 11 (14%) and 6 (8%) articles, respectively. In total, 38 articles (48%) reported that data for all participants were included in the analysis and 18 (23%) that some participants were excluded from analysis; the remaining 23 (29%) gave no information on potential exclusion. The statistical analysis test was reported in 73 articles (94%) and was ANOVA for 67 articles (86%) and *t* test for 6 (8%). Six articles (6%) did not report which test was used. For the 67 articles reporting ANOVA, 78% described taking into account period, 73% sequence, 66% formulation and 66% subject within sequence.

### Setting and population characteristics

As shown in Table S3, the location of the study was reported in 44 articles (56%). Of these, 22 (50%) studies were performed in Asia. In total, 73 articles (92%) described the inclusion of exclusively healthy participants. Six articles about NTI drugs (38%) described the inclusion of non-healthy participants. The age of participants was reported in 62 articles (78%), and the median age was 28 years (IQR 23 to 33). Exclusion criteria were reported in 86% of articles and were mainly concomitant medication use (57%), significant psychiatric or medical disease (56%) and positivity for hepatitis B or C virus (50%) and HIV (40%).

### Results and conclusions for single-dose bioequivalence trials (Table S4)

**Figure 2:**
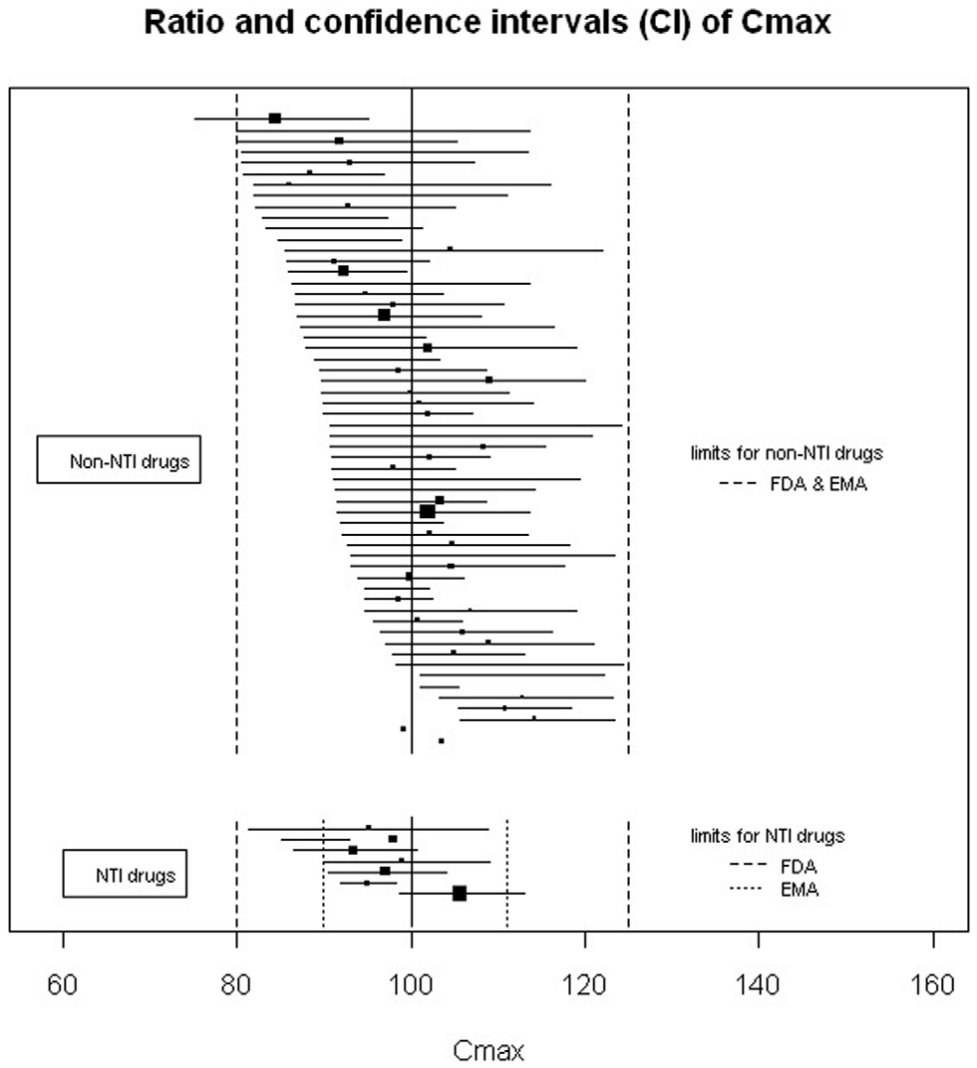
Ratio and 90% confidence intervals for the C_max_ of single-dose bioequivalence trials with a conclusion of bioequivalence. Black squares indicate ratio; horizontal lines, 90% CI. The size of each square reflects the number of participants enrolled. Limits of acceptance required by both FDA and EMA for confidence intervals of the ratio are within 80% and 125% of the ratio of the generic to brand-name drug.

**Figure 3:**
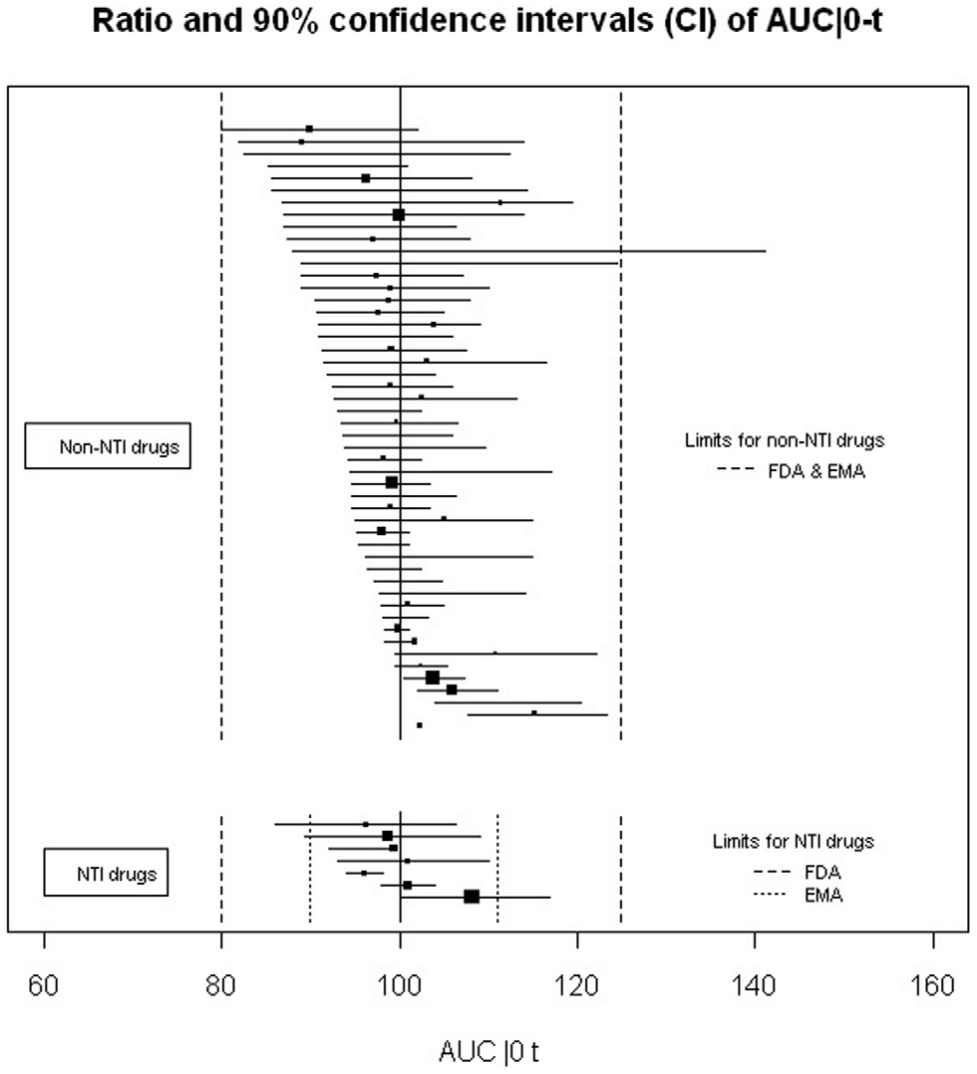
Ratio and 90% confidence intervals for the AUC|_0 to t_ of single-dose bioequivalence trials with a conclusion of bioequivalence. Black squares indicate ratio; horizontal lines, 90% CI. The size of each square reflects the number of participants enrolled. Limits of acceptance required by both the US Food and Drug Administration (FDA) and the European Medicine Agency (EMA) for confidence intervals of the ratio are within 80% and 125% of the ratio for the generic to brand-name drug.

**Figure 4:**
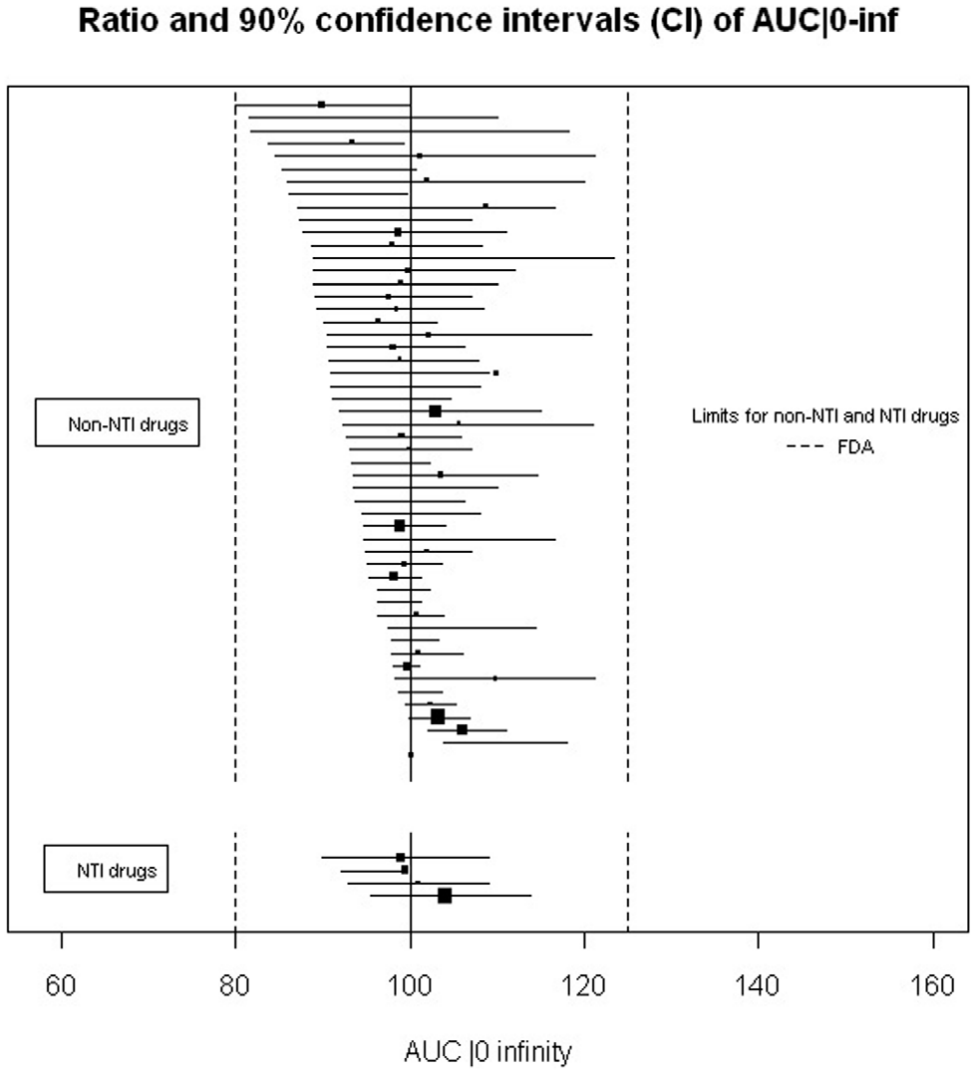
Ratio and 90% confidence intervals for the AUC|_0 to infinity_ of single-dose bioequivalence trials with a conclusion of bioequivalence. Black squares indicate ratio; horizontal lines, 90% CI. The size of each square reflects the number of participants enrolled. Limits of acceptance required by FDA for confidence intervals of the ratio are within 80% and 125% of the ratio of the generic to brand-name drug.

Among the reports of single-dose trials (n = 73), for 65 (89%) the conclusion was bioequivalence, for 7 (10%) non-bioequivalence, and for one, the conclusion was unclear. For the articles with bioequivalence conclusion, 45 (69%) reported 90% CIs within the limits required for the 3 criteria as required by the FDA, 15 (23%) reported appropriate 90% CIs for 2 criteria, 8 of which reported both C_max_ and AUC|_0 to t_ as required by the EMA, and 2 (2%) did not report any 90% CIs for the criteria. Two articles (3%) with bioequivalence conclusions described 90% CIs outside the limits. In one trial, the 90% CI for C_max_ for the ratio was between 77% and 133%, which is authorized by EMA for highly variable drugs (i.e., drugs exhibiting intra-subject variability greater than 30%). Figures 2, 3 and 4 show the details of the 90% CIs for the ratio of generic to brand-name for C_max_, AUC|_0 to t_ and, AUC|_0 to infinity_ of generic to brand-name drugs for all reports of single-dose trials with bioequivalence conclusions separately for NTI and non-NTI drugs.

## Discussion

Generic drugs are used by many patients for economic reasons, so their evaluation must be highly transparent. We systematically reviewed the quality of reporting of bioequivalence trials comparing generic to brand-name drugs and found it to be overall poor. In 41% of articles, the name of the reference drug was not given. Information was insufficient for funding source, important items for assessing the risk of bias within trials and country where the trial was performed. More importantly, a substantial number of articles of single-dose trials concluding bioequivalence did not report the 90% CI for the criteria required by the Health authorities.

The objectives and methodology of bioequivalence trials differ greatly from those of noninferiority and equivalence trials. Bioequivalence trials are usually randomized crossover trials including a small sample of healthy volunteers aiming to demonstrate that 2 molecules are chemically bioequivalent on the basis of pharmacokinetic criteria: rate of absorption as determined by the peak plasma concentration (Cmax), area under the plasma concentration–time curve from time 0 to time t = end of the study (AUC|0 to t) and to infinity (AUC|0 to infinity). Limits used to conclude bioequivalence are fixed by regulatory agencies. Noninferiority and equivalence trials aim to demonstrate that the experimental treatment is not clinically different from the comparator (an active control treatment) by more than a pre-specified small amount known as the equivalence margin and which is fixed by the investigators of the trial and varies greatly among trials. Equivalence trials are usually parallel-group trials including a large number of patients (the smaller the margin, the larger the number of patients) and clinical outcomes. Some previous methodological reviews [16], [17] assessed the quality of reporting for noninferiority and/or equivalence trials and showed important deficiencies, but to our knowledge, this is the first review focusing on bioequivalence trials.

Substituting brand-name for generic for drugs is crucial for reducing healthcare costs. As an example, in the United States, the use of generic drugs has saved the healthcare system more than $734 billion between 1999 and 2008, with approximately $121 billion in savings in 2008 alone. So, the development of generic drugs is important. Because this development leads to the use of generics by millions of patients, the evaluation of these drugs must be highly transparent, with registration of trials, publication of results of registered trials and adequate reporting of results. Our aim was not to discuss the methodology of trials assessing generic drugs but to determine whether the variables currently recommended to conclude bioequivalence were adequately reported.

Only one article reported a trial's registration number in an international database. Given the number of existing generic drugs, it is likely that bioequivalence data are not published for most of them. During the study period, 1,661 new generic drugs were approved by the FDA [18]. Reports of trials assessing generic drugs were not available on FDA or EMA websites. Moreover, our sample of published reports of bioequivalence trials included mostly positive bioequivalence trial results. So publication bias [19] may also affect bioequivalence trials comparing generic to brand-name drugs because it is not likely that only 10% of trials failed to demonstrate bioequivalence.

We found a high number of articles (55) that did not give the name of the reference drug, so we could not ascertain whether the reference drug was the brand-name or another generic drug. The readers need to know which comparator was used because equivalence trials require the efficacy of the reference treatment to be well established [20], [21]. If the reference drug is not the brand-name drug, bio-creep [20] leading to a significant loss of activity compared to the brand-name drug could occur, as was shown in 2 simulation studies [22], [23]. The consequences can be important for patients, especially for drugs with an NTI, because small changes in systemic concentration can lead to substantial changes in clinical response and toxicity of the drug.

Our results also show lack of transparency in published reports of trials. Important information such as funding source, country where the trial was performed and details about the methodology were frequently missing. Knowing which population was used and how the trial was performed (i.e., randomization method, whether data for some participants were excluded from analysis) is essential to critically judge whether the results are valid and reproducible. More importantly, about one-third of articles of single-dose trials concluding bioequivalence did not report adequately 90% CIs for the 3 criteria required by the FDA. As the EMA required only two of these criteria, also about 20% of these articles did not report these criteria. If the required information to conclude bioequivalence is missing, readers cannot judge whether the conclusion is reliable.

Our study has some limitations. The search period was short, 2005 to 2008. Nevertheless, our study relied on guidelines published relatively recently by the FDA, in 2003, and by the EMA, in 2001 and updated in 2008. We used PubMed only to identify relevant trials, so our search may not have been exhaustive because of incomplete journal coverage. Embase covers pharmacology journals well and has indexing terms suited to this topic, so future reviews should also involve searching this database. However, the search strategy we used to obtain articles revealed only 1 additional article in Embase that could have been included in our study. Our sample included few articles of bioequivalence trials performed in Europe or North America. We do not have any satisfactory explanation for this situation.

In conclusion, because generic drugs are necessary and are used by many people, we argue for increased transparency of published results of trials comparing generic to brand-name drugs. Efforts should be on improving the reporting of bioequivalence data with registration of trial protocols and publication of trial results. The reference drug used should be systematically documented as the drug with demonstrated efficacy. Important information for judging both internal and external validity should be systematically reported, as should the 90% CIs for the 3 criteria (mean C_max_ , AUC|_0 to t_, and AUC|_0 to infinity_) for the ratio of generic to brand-name drug required by the FDA, so that readers can judge the reliability of the conclusions.

## Supporting Information

Table S1 General characteristics of bioequivalence trials comparing generic to brand-name drugs according to narrow therapeutic index (NTI) of the drugs (n = 79 reports).
(DOC)

Table S2 Study design and methodology of bioequivalence studies comparing generic to brand-name drugs according to narrow therapeutic index (NTI) of the drugs (n = 79 reports).
(DOC)

Table S3 Reporting of setting and population characteristics of bioequivalence studies comparing generic to brand-name drugs according to narrow therapeutic index (NTI) of the drugs (n = 79 reports).
(DOC)

Table S4 Synthesis of the results according to the conclusions reported for the single-dose bioequivalence studies comparing generic to brand-name drugs according to narrow therapeutic index (NTI) of the drugs (n = 73 reports).
(DOC)

Appendix S1 Bioequivalence studies assessing non-narrow therapeutic index drugs.
(DOC)

Appendix S2 Bioequivalence studies assessing narrow therapeutic index drugs.
(DOC)

Appendix S3 Studies excluded because the name of the reference drug was not clearly reported.
(DOC)

Appendix S4 PRISMA checklist.
(DOC)
