# Supplementary material for: Quality of Reporting of Bioequivalence Trials Comparing Generic to Brand Name Drugs: A Methodological Systematic Review
Source: PLoS One. 2011 Aug 17;6(8):e23611. doi: 10.1371/journal.pone.0023611 (PMC3157430; doi:10.1371/journal.pone.0023611)
Supplement: Table S1 — General characteristics of bioequivalence trials comparing generic to brand-name drugs according to narrow therapeutic index (NTI) of the drugs (n = 79 reports). (DOC) [file pone.0023611.s001.doc]

Table S1: General characteristics of bioequivalence trials comparing generic to brand-name drugs according to narrow therapeutic index (NTI) of the drugs (n=79 reports)

| **Characteristics** | **Overall**  **N=79**  **n (%)** | **Non-NTI**  **N=63**  **n (%)** | **NTI**  **N=16**  **n (%)** |
| --- | --- | --- | --- |
| **Type of journal of publication** |  |  |  |
| Pharmacology  General  Speciality | 62 (78)  9 (12)  8 (10) | 51 (81)  8 (13)  4 (6) | 11 (69)  1 (6)  4 (25) |
| **Funding source** |  |  |  |
| Reported  Private  Public  Both public and private  Not reported | 32 (41)  20 (25)  7 (9)  5 (6)  47 (59) | 23 (36)  13 (21)  5 (8)  5 (8)  40 (63) | 9 (56)  7 (44)  2 (12)  0  7 (44) |
| **Drug class** |  |  |  |
| Anti-infectives for systemic use  Cardiovascular system  Nervous system  Antineoplasic and immunomodulating agents  Alimentary tract and metabolism  Musculo-skeletal system  Systemic hormonal preparations  Respiratory system  Antiparasitic products  Genito-urinary system and sex hormones  Sensory organs | 29 (37)  14 (18)  11 (14)  9 (11)  9 (11)  4 (5)  1 (1)  2 (2)  0  0  0 | 24 (38)  14 (22)  9 (14)  1 (2)  9 (14)  4 (6)  1 (2)  1 (2)  0  0  0 | 5 (31)  0  2 (12)  8 (50)  0  0  0  1 (6)  0  0  0 |
| **Administration of the drug** |  |  |  |
| **Dose**  Single  Multiple  **Fasting**  Yes  No  **Route**  Oral  Parenteral  Topical | 73 (92)  6 (8)  66 (83)  13 (17)  76 (96)  3 (4)  0 | 63 (100)  0  55 (87)  8 (13)  60 (95)  3 (5)  0 | 10 (62)  6 (38)  11 (69)  5 (31)  16 (100)  0  0 |
