# Supplementary material for: Quality of Reporting of Bioequivalence Trials Comparing Generic to Brand Name Drugs: A Methodological Systematic Review
Source: PLoS One. 2011 Aug 17;6(8):e23611. doi: 10.1371/journal.pone.0023611 (PMC3157430; doi:10.1371/journal.pone.0023611)
Supplement: Table S2 — Study design and methodology of bioequivalence studies comparing generic to brand-name drugs according to narrow therapeutic index (NTI) of the drugs (n = 79 reports). (DOC) [file pone.0023611.s002.doc]

Table S2: Study design and methodology of bioequivalence studies comparing generic to brand-name drugs according to narrow therapeutic index (NTI) of the drugs (n=79 reports)

|  | **Overall**  **N=79**  **n (%)** | **Non-NTI**  **N=63**  **n (%)** | **NTI**  **N=16**  **n (%)** |
| --- | --- | --- | --- |
| **Registration number in an international database** |  |  |  |
| Reported  Not reported | 1 (1)  78 (99) | 0  63 (100) | 1 (6)  15 (94) |
| **Study design** |  |  |  |
| **Reported**  Randomized controlled trial  Cross-over design  **Not reported** | 78 (99)  78 (99)  78 (99)  1 (1) | 62 (98)  62 (98)  62 (98)  1 (2) | 16(100)  16 (100)  16(100)  0 (0) |
| Sample size |  |  |  |
| Sample size, median (interquartile range)  Sample size calculation reported  Details of sample size calculation reported | 24 (24–28)  27 (34)  16 (20) | 24 (24–28)  18 (29)  13 (21) | 24 (19–36)  9 (56)  3 (18) |
| **Internal validity of studies** |  |  |  |
| **Method used to generate randomization sequence**  Adequate  Inadequate  Not reported  **Allocation concealment**  Adequate  Inadequate  Not reported  **Blinding of study participants**  **Blinding of care providers**  **Blinding of outcome assessors**  **Complete outcome data analysis**  Yes, analysis of data for all study participants reported  No, exclusion of participants from analysis reported  Not reported | 12 (15)  0  67 (85)  4 (5)  0  75 (95)  13 (16)  11 (14)  6 (8)  38 (48)  18 (23)  23 (29) | 7 (11)  0  56 (89)  3 (5)  0  60 (95)  11 (17)  9 (14)  5 (8)  32 (50)  13 (21)  18 (29) | 5 (31)  0  11 (69)  1 (6)  0  15 (94)  2 (12)  2 (12)  1 (6)  6 (38)  5 (31)  5 (31) |
| **Statistical analysis** |  |  |  |
| **Reported**  **ANOVA**  Sequence variance  Subject within sequence variance  Period variance  Formulation variance  ***t* test**  **Not reported** | 73 (92)  67 (92)  50 (75)  45 (67)  53 (79)  45 (67)  6 (8)  6 (6) | 59 (94)  57 (97)  42 (74)  39 (68)  45 (79)  38 (67)  2 (3)  4 (6) | 14 (87)  10 (71)  8 (80)  6 (60)  8 (80)  7 (70)  4 (29)  2 (13) |
