# Supplementary material for: Quality of Reporting of Bioequivalence Trials Comparing Generic to Brand Name Drugs: A Methodological Systematic Review
Source: PLoS One. 2011 Aug 17;6(8):e23611. doi: 10.1371/journal.pone.0023611 (PMC3157430; doi:10.1371/journal.pone.0023611)
Supplement: Table S3 — Reporting of setting and population characteristics of bioequivalence studies comparing generic to brand-name drugs according to narrow therapeutic index (NTI) of the drugs (n = 79 reports). (DOC) [file pone.0023611.s003.doc]

Table S3: Reporting of setting and population characteristics of bioequivalence studies comparing generic to brand-name drugs according to narrow therapeutic index (NTI) of the drugs (n=79 reports)

|  | **Overall**  **n=79**  **n (%)** | **Non-NTI**  **n=63**  **n (%)** | **NTI**  **n=16**  **n (%)** |
| --- | --- | --- | --- |
| **Reporting of the geographical location of the study** | **44 (56)** | **35 (56)** | **9 (56)** |
| Asia  Middle East  South America  Africa  Europe  North America  Oceania | 22 (50)  9 (20)  7 (16)  3 (7)  2 (5)  1 (2)  0 | 19 (54)  9 (26)  5 (14)  0  2 (6)  0  0 | 3 (33)  0  2 (23)  3 (33)  0  1 (11)  0 |
| **Characteristics of the study participants** |  |  |  |
| **Health status**  Healthy  Not healthy  **Age**  Median age, years (interquartile range)  Not reported  **Sex**  Studies including men only  Not applicable (drug used for only one sex)  Studies not reporting the proportion of males to females  **Body mass index**  20–25  >25  Not reported | 73 (92)  6 (8)  28 (23–33)  17 (22)  35 (44)  1 (1)  7 (9)  28 (35)  4 (5)  47 (59) | 63 (100)  0  26 (23–31)  15 (24)  31 (49)  1 (2)  7 (11)  22 (35)  2 (3)  39 (62) | 10 (62)  6 (38)  36 (27–38)  2 (12)  4 (25)  0  0  6 (38)  2 (13)  8 (50) |
| **Reporting of exclusion criteria for the study participants** | **68 (86)** | **54 (86)** | **14 (87)** |
| Concomitant medication use  Significant psychiatric or medical disease  Positive hepatitis B or C test result  Positive HIV test result  Drug hypersensitivity  Weight  Tobacco use  Alcohol consumption  Substance abuse  Height  Age  Recent participation in another trial  Other | 39 (57)  38 (56)  34 (50)  27 (40)  27 (40)  23 (34)  22 (32)  22 (32)  15 (22)  14 (21)  13 (19)  5 (7)  35 (51) | 35 (65)  32 (59)  29 (54)  23 (43)  25 (46)  14 (26)  18 (33)  18 (33)  11 (20)  10 (19)  7 (13)  4 (7)  22 (41) | 4 (29)  6 (43)  5 (36)  4 (29)  2 (14)  9 (64)  4 (29)  4 (29)  4 (29)  4 (29)  6 (43)  1 (7)  13 (93) |
