# Supplementary material for: Quality of Reporting of Bioequivalence Trials Comparing Generic to Brand Name Drugs: A Methodological Systematic Review
Source: PLoS One. 2011 Aug 17;6(8):e23611. doi: 10.1371/journal.pone.0023611 (PMC3157430; doi:10.1371/journal.pone.0023611)
Supplement: Table S4 — Synthesis of the results according to the conclusions reported for the single-dose bioequivalence studies comparing generic to brand-name drugs according to narrow therapeutic index (NTI) of the drugs (n = 73 reports). (DOC) [file pone.0023611.s004.doc]

Table S4: Synthesis of the results according to the conclusions reported for the single-dose bioequivalence studies comparing generic to brand-name drugs according to narrow therapeutic index (NTI) of the drugs (n=73 reports)

| **Conclusion reported** | **n (%) N=73** | **Non-NTI**  **N= 63** | **NTI**  **N= 10** |
| --- | --- | --- | --- |
| **Bioequivalence** | **65 (89)** | **58 (92)** | **7 (70)** |
| **90% CIs for the 3 criteria reported**  Within 80-125% for all 3 criteria  Within 80-125% for AUC|_0 to t_, AUC|_0 to infinity_  and within 77%-133% for C_max_^$^  Within 80-125% for AUC|_0 to t_, AUC|_0 to infinity_ and outside limits for C_max_  Within 80-125% for AUC|_0 to infinity_ and C_max_ and outside limits for AUC|_0 to t_,  **90% CIs for 2 criteria reported**  AUC|_0 to t_, and C_max_ reported and within 80-125%  AUC|_0 to infinity_ and C_max_ reported and within 80-125%  **No confidence intervals reported** | **48 (74)**  45 (69)  1 (1)  1 (1)  1 (1)  **15 (23)**  8 (12)  7 (11)  **2 (3)** | **44 (76)**  41 (70)  1 (2)  1 (2)  1 (2)  **12 (21)**  5 (9)  7 (12)  **2(3)** | **4 (57)**  4 (57)  0 (0)  0 (0)  0 (0)  **3 (43)**  3 (43)  0 (0)  **0 (0)** |
| Non-bioequivalence | **7 (10)** | **4 (6)** | **3 (30)** |
| **90% CIs for the 3 criteria reported**  AUC|_0 to t_, AUC|_0 to infinity_ and C_max_ outside 80-125%  C_max_ outside 80-125%  **90% CIs for 2 criteria reported**  AUC|_0 to t_, C_max_ outside 80-125%  AUC|_0 to t_  outside 80-125% | **5 (71)**  4 (57)  1 (14)  **2 (29)**  1 (14)  1 (14) | **4 (100)**  3 (75)  1 (25)  **0 (0)**  0 (0)  0 (0) | **1 (33)**  1 (33)  **2 (67)**  1 (33)  1 (33) |
| **Conclusion unclear** | **1 (1)** | **1 (2)** | **0 (0)** |
| **90% CI of the ratio for the 3 criteria reported:**  AUC|_0 to t_, AUC|_0 to infinity_ and C_max_ outside 80-125% |  |  |  |

^$^ Limits authorized for highly variable drugs (i.e., drugs exhibiting intra-subject variability greater than 30%) by the European Medicine Agency
