# Supplementary material for: Quality of Reporting of Bioequivalence Trials Comparing Generic to Brand Name Drugs: A Methodological Systematic Review
Source: PLoS One. 2011 Aug 17;6(8):e23611. doi: 10.1371/journal.pone.0023611 (PMC3157430; doi:10.1371/journal.pone.0023611)
Supplement: Appendix S1 — Bioequivalence studies assessing non-narrow therapeutic index drugs. (DOC) [file pone.0023611.s005.doc]

Appendix S1: Bioequivalence studies assessing non-narrow therapeutic index drugs

| **Study** | **Journal** | **Generic Drug/ BRAND NAME DRUG** | **Primary outcome** | **Population** | **Study design** | **Result** |
| --- | --- | --- | --- | --- | --- | --- |
| Al-Gaai E  2005 | Biopharm  Drugs Dispos | Fluconazole  DIFLUCAN | AUC|_0 t_  AUC|_0 infinity_  C_max_ | 28  healthy  males | RCT  Cross-over  Single dose | Bioequivalence |
| Al-Mohzea AM 2007 | Int J Clin  Pharmacol Ther | Gemifloxacin  FACTIVE | AUC|_0 t_  AUC|_0 infinity_  C_max_ | 24  healthy  males | RCT  Cross-over  Single dose | Bioequivalence |
| Amini H  2008 | Clin Drug  Investig | Acyclovir  ZOVIRAX | AUC|_0 t_  AUC|_0 infinity_  C_max_ | 12  healthy  males | RCT  Cross-over  Single dose | Bioequivalence not proved |
| Asiri YA  2005 | Int J Clin  Pharmacol Ther | Cefixime  SUPRAX | AUC|_0 t_  AUC|_0 infinity_  C_max_ | 24  healthy  males | RCT  Cross-over  Single dose | Bioequivalence |
| Baglie S  2005 | Int J Clin  Pharmacol Ther | Amoxicillin  AMOXIL BD | AUC|_0 t_  AUC|_0 infinity_  C_max_ | 26  healthy  males/females | RCT  Cross-over  Single dose | Bioequivalence |
| Boonleang J  2007 | Clin Ther | Azithromycin  ZITHROMAX | AUC|_0 t_  AUC|_0 infinity_  C_max_ | 12  healthy  males/females | RCT  Cross-over  Single dose | Bioequivalence |
| Bramlage P  2008 | BMC  Pharmacol | Ibuprofen  NUROFEN | AUC|_0 t_  AUC|_0 infinity_  C_max_ | 60  healthy  males/females | RCT  Cross-over  Single dose | Bioequivalence |
| Chatsiricharoenkul S,  2007 | J Med  Assoc Thai | Glista  ACTOS | AUC|_0 t_  AUC|_0 infinity_  C_max_ | 24  healthy  males/females | RCT  Cross-over  Single dose | Bioequivalence |
| Cho HY  2006 | Int J Clin  Pharmacol Ther | Gabatin  NEURONTIN | AUC|_0 t_  AUC|_0 infinity_  C_max_ | 26  healthy  males | RCT  Cross-over  Single dose | Bioequivalence |
| Chompootaweep S  2006 | J Med  Assoc Thai | Antivir  RETROVIR | AUC|_0 t_  AUC|_0 infinity_  C_max_ | 28  healthy  males | RCT  Cross-over  Single dose | Bioequivalence |
| Dos Reis Serra  2008 | Clin Ther | Zidovudina  RETROVIR | AUC|_0 t_  AUC|_0 infinity_  C_max_ | 24  healthy  males/females | RCT  Cross-over  Single dose | Bioequivalence |
| Eradiri O  2007 | Curr Med  Res Opin | Tramadolor  ULTRAM | C_max_  AUC_ζ_ | 48  healthy  males/females | RCT  Cross-over  Multiple dose | Bioequivalence |
| Flores-Murrieta FJ  2006 | Proc West  Pharmacol Soc | Gen-Glybe  DAONIL | AUC|_0 t_  AUC|_0 infinity_  C_max_ | 24  healthy  males | RCT  Cross-over  Single dose | Bioequivalence |
| Flores-Murrieta FJ  2008 | Biopharm  Drug Dispos | Midaven  AZANTAC | AUC|_0 t_  C_max_ | 25  healthy  females | RCT  Cross-over  Single dose | Bioequivalence |
| Hassan Y  2007 | Singapore  Med J | Ciprofloxacin  CIPROBAY | AUC|_0 infinity_  C_max_ | 24  healthy | RCT  Cross-over  Single dose | Bioequivalence |
| Hernandez-Bernal F  2005 | Biopharm  Drug Dispos | Herbervital  NEUPOGEN | AUC|_0 t_  AUC|_0 infinity_  C_max_ | 24  healthy  males | RCT  Cross-over  Single dose | Bioequivalence |

| **Study** | **Journal** | **Generic Drug/ BRAND NAME DRUG** | **Primary outcome** | **Population** | **Study design** | **Result** |
| --- | --- | --- | --- | --- | --- | --- |
| Jiang X  2008 | Arzneimittel  forschung | Terbinafine | AUC|_0 t_  AUC|_0 infinity_  C_max_ | 20  healthy  males | RCT  Cross-over  Single dose | Bioequivalence |
| Jovanovic D  2006 | J Clin Pharmacol | Bisoprolol  CONCOR | AUC|_0 infinity_  C_max_ | 24  healthy  males/females | RCT  Cross-over  Single dose | Bioequivalence |
| Jovanovic D  2006 | Pharmazie | Bronchobos MUCOPRONT | AUC|_0 infinity_  C_max_ | 18  healthy  males/females | RCT  Cross-over  Single dose | Bioequivalence |
| Jovanovic D  2005 | Clin Ther | Funzol  DIFLUCAN | AUC|_0 infinity_  C_max_ | 24  healthy  males/females | RCT  Cross-over  Single dose | Bioequivalence |
| Jovanovic D  2006 | Eur J Drug  Metab  Pharmacokinet | Fluoxetin  PROZAC | AUC|_0 infinity_  C_max_ | 24  healthy  males/females | RCT  Cross-over  Single dose | Bioequivalence |
| Jovanovic D  2006 | Vojnosanit  Pregl | Glimepirid  AMARYL | AUC|_0 infinity_  C_max_ | 24  healthy  males/females | RCT  Cross-over  Single dose | Bioequivalence |
| Kaehler ST  2006 | Pharmacology | Desmopressin  MINIRIN | AUC|_0 t_  AUC|_0 infinity_  C_max_ | 60  healthy  males/females | RCT  Cross-over  Single dose | Bioequivalence |
| Kongpatanakul S  2008 | Int J Clin  Pharmacol Ther | GPO A flu  TAMIFLU | AUC|_0 t_  AUC|_0 infinity_  C_max_ | 24  healthy | RCT  Cross-over  Single dose | Bioequivalence |
| Leelarasamee A  2008 | J Med  Assoc Thai | Mapenem  MERONEN | AUC|_0 t_  AUC|_0 infinity_  C_max_ | 26  nonhealthy  males | RCT  Cross-over  Single dose | Bioequivalence |
| Leelarasamee A  2006 | J Med  Assoc Thai | ClinottP  DALACIN | AUC|_0 t_  AUC|_0 infinity_  C_max_ | 24  healthy  males | RCT  Cross-over  Single dose | Bioequivalence |
| Li J  2008 | Arzneimittel  forschung | Clindamycin | AUC|_0 t_  AUC|_0 infinity_  C_max_ | 24  healthy  males | RCT  Cross-over Single dose | Bioequivalence |
| Lockyer M  2005 | Biopharm  Drug Dispos | Nizax  AXID | AUC|_0 t_  AUC|_0 infinity_  C_max_ | 20  healthy  males | RCT  Cross-over Single dose | Bioequivalence |
| Mahatthanatrakul W  2008 | Int J Clin  Pharmacol Ther | Quantia 200  SEQUOREL | AUC|_0 t_  AUC|_0 infinity_  C_max_ | 24  healthy  males | RCT  Cross-over Single dose | Bioequivalence |
| Marcelin-Jimenez G  2005 | Biopharm  Drug Dispos | Promotion  MOBICOX | AUC|_0 t_  AUC|_0 infinity_  C_max_ | 24  healthy  females | RCT  Cross-over Single dose | Bioequivalence |
| Marier JF  2006 | Int J Clin  Pharmacol Ther | Abacavir  ZIAGEN | AUC|_0 t_  AUC|_0 infinity_  C_max_ | 40  healthy  males | RCT  Cross-over Single dose | Bioequivalence |
| Marier JF  2006 | Int J Clin  Pharmacol Ther | Zidovudine  RETROVIR | AUC|_0 t_  AUC|_0 infinity_  C_max_ | 68  healthy  males/females | RCT  Cross-over Single dose | Bioequivalence |
| Mendes GD  2005 | Int J Clin  Pharmacol Ther | Citalopram  CIPRAMIL | AUC|_0 t_  AUC|_0 infinity_  C_max_ | 26  healthy  males/females | RCT  Cross-over Single dose | Bioequivalence |
| Mendes GD  2005 | Int J Clin  Pharmacol Ther | Citalopram  CIPRAMIL | AUC|_0 t_  AUC|_0 infinity_  C_max_ | 26  healthy  males/females | RCT  Cross-over Single dose | Bioequivalence |
| Mendes GD  2006 | Int J Clin  Pharmacol Ther | Ramipril  TRIATEC | AUC|_0 t_  C_max_ | 26  healthy  males/females | RCT  Cross-over Single dose | Bioequivalence |

| **Study** | **Journal** | **Generic Drug/ BRAND NAME DRUG** | **Primary outcome** | **Population** | **Study design** | **Result** |
| --- | --- | --- | --- | --- | --- | --- |
| Mendes GD  2008 | Int J Clin  Pharmacol Ther | Ibuprofen  ADVIL/ ALIVIUM | AUC|_0 t_  AUC|_0 infinity_  C_max_ | 24  healthy  males/females | RCT  Cross-over Single dose | Bioequivalence |
| Mendes GD  2007 | Int J Clin  Pharmacol Ther | Gliclazide  DIAMICRON | AUC|_0 t_  AUC|_0 infinity_  C_max_ | 30  healthy  males/females | RCT  Cross-over Single dose | Bioequivalence  not proven |
| Mendoza L  2006 | Pharmazie | Divator  LIPITOR | AUC|_0 t_  AUC|_0 infinity_  C_max_ | 52  healthy  males | RCT  Cross-over Single dose | Bioequivalence |
| Mignini F  2007 | Clin Exp  Hypertens | Tioctil N  THIOCTACID 600 HR | AUC|_0 t_  C_max_ | 32  healthy  males/females | RCT  Cross-over Single dose | Bioequivalence |
| Mignini F  2008 | Clin Exp  Hypertens | Lovastatin  MEVINACOR | AUC|_0 t_  AUC|_0 infinity_  C_max_ | 36  healthy  males/females | RCT  Cross-over Single dose | Bioequivalence |
| Mignini F  2007 | Clin Exp  Hypertens | Amlodipine Maleate  NORVASC | AUC|_0 t_  AUC|_0 infinity_  C_max_ | 24  healthy  males/females | RCT  Cross-over Single dose | Bioequivalence |
| Monif T  2007 | Int J Clin  Pharmacol Ther | Stavudine  ZERIT | AUC|_0 t_  AUC|_0 infinity_  C_max_ | 40  healthy  males | RCT  Cross-over Single dose | Bioequivalence |
| Najib NM  2005 | Biopharm Drug Dispos | Tensotin  TENORMIN | AUC|_0 t_  AUC|_0 infinity_  C_max_ | 24  healthy  males | RCT  Cross-over Single dose | Bioequivalence |
| Najib NM  2005 | Biopharm Drug Dispos | Lovrak  ZOVIRAX | AUC|_0 t_  AUC|_0 infinity_  C_max_ | 24  healthy  males | Cross-over Single dose | Bioequivalence |
| Najib NM  2005 | Biopharm Drug Dispos | Flutin  PROZAC | AUC|_0 t_  AUC|_0 infinity_  C_max_ | 24  healthy  males | RCT  Cross-over Single dose | Bioequivalence |
| Oliveira CH  2006 | Int J Clin  Pharmacol Ther | Losartan  COZAAR | AUC|_0 t_  AUC|_0 infinity_  C_max_ | 26  healthy  males/females | RCT  Cross-over Single dose | Bioequivalence |
| Park JY  2006 | Int J Clin  Pharmacol Ther | Naidipine  NORVASC | AUC|_0 t_  AUC|_0 infinity_  C_max_ | 18  healthy  males | RCT  Cross-over Single dose | Bioequivalence |
| Pineyro-Lopez A,  2007 | Clin Ther | Macrozit  AZITROCIN | AUC|_0 t_  AUC|_0 infinity_  C_max_ | 28  healthy  males/females | RCT  Cross-over Single dose | Bioequivalence |
| Pistos C  2005 | Int J Clin  Pharmacol Ther | Glimepiride  SOLOSA | AUC|_0 t_  AUC|_0 infinity_  C_max_ | 24  healthy  males/females | RCT  Cross-over Single dose | Bioequivalence |
| Porta V  2005 | Int J Pharm | Flunazole  ZOLTEC | AUC|_0 t_  AUC|_0 infinity_  C_max_ | 28  healthy  males/females | RCT  Cross-over Single dose | Bioequivalence |
| Rojanasthien N  2008 | J Med  Assoc Thai | Quinaril  ACCUPRIL | AUC|_0 t_  AUC|_0 infinity_  C_max_ | 24  healthy  males | RCT  Cross-over Single dose | Bioequivalence |
| Ruenis AP  2005 | Int J Clin  Pharmacol Ther | Clarythromicina  KLARICID | AUC|_0 t_  AUC|_0 infinity_  C_max_ | 24  healthy | RCT  Cross-over Single dose | Bioequivalence |
| Sailer R,  2007 | Arzneimittel  forschung | amlodipine | AUC|_0 t_  AUC|_0 infinity_  C_max_ | 18  healthy  males | RCT  Cross-over Single dose | Bioequivalence |
| Srichaiya A  2008 | Clin Ther | Lamidus  LAMICTAL | AUC|_0 t_  AUC|_0 infinity_  C_max_ | 24  healthy  males | RCT  Cross-over Single dose | Bioequivalence |
| Sripalakit P  2007 | Drug Dev  Ind Pharm | Glubosil  ACTOS | AUC|_0 t_  AUC|_0 infinity_  C_max_ | 35  healthy  Males | RCT  Cross-over Single dose | Bioequivalence |

| **Study** | **Journal** | **Generic Drug/ BRAND NAME DRUG** | **Primary outcome** | **Population** | **Study design** | **Result** |
| --- | --- | --- | --- | --- | --- | --- |
| Tamimi JJ  2005 | Biopharm  Drug Dispos | Lisotec  ZESTRIL | AUC|_0 t_  AUC|_0 infinity_  C_max_ | 28  healthy  males | RCT  Cross-over Single dose | Bioequivalence |
| Tamimi JJ  2005 | Biopharm  Drug Dispos | Blozart  COZAAR | AUC|_0 t_  AUC|_0 infinity_  C_max_ | 24  healthy  males | RCT  Cross-over Single dose | Bioequivalence |
| Sripalakit P  2005 | Drug Dev  Ind Pharm | Dozozin 2  CARDURA | AUC|_0 t_  AUC|_0 infinity_  C_max_ | 24  healthy  males | RCT  Cross-over Single dose | Bioequivalence |
| van Os S  2007 | Int J Clin  Pharmacol Ther | Risperidone  RISPERDAL | AUC|_0 t_  AUC|_0 infinity_  C_max_ | 28  healthy  males/female | RCT  Cross-over Single dose | Bioequivalence  not proven |
| Vezina HE  2006 | J Acquir  Immune  Defic Syndr | Duovir  COMBIVIR | AUC|_0 t_  AUC|_0 infinity_  C_max_ | 15  healthy  females | RCT  Cross-over Single dose | Bioequivalence  Not proven |
| Xu FG,  2008 | Arzneimittel  forschung | Spironolactone | AUC|_0 t_  AUC|_0 infinity_  C_max_ | 20  healthy  males | RCT  Cross-over Single dose | Bioequivalence |
| Yun MH  2006 | Arch Pharm  Res | Alenmax  FOSAMAX | AUC|_0 t_  AUC|_0 infinity_  C_max_ | 20  healthy | RCT  Cross-over Single dose | Bioequivalence |
| Zaid AN  2008 | Arzneimittel  forschung | cedfinir | AUC|_0 t_  AUC|_0 infinity_  C_max_ | 24  healthy  males | RCT  Cross-over Single dose | Bioequivalence |
| Zaid AN  2006 | Int J Clin Pharmacol Ther | Fluxicare  PROZAC | AUC|_0 t_  AUC|_0 infinity_  C_max_ | 24  healthy  males | RCT  Cross-over Single dose | Bioequivalence |

RCT=randomized controlled trial
