# Supplementary material for: Quality of Reporting of Bioequivalence Trials Comparing Generic to Brand Name Drugs: A Methodological Systematic Review
Source: PLoS One. 2011 Aug 17;6(8):e23611. doi: 10.1371/journal.pone.0023611 (PMC3157430; doi:10.1371/journal.pone.0023611)
Supplement: Appendix S2 — Bioequivalence studies assessing narrow therapeutic index drugs. (DOC) [file pone.0023611.s006.doc]

Appendix S2: Bioequivalence studies assessing narrow therapeutic index drugs

| **Study** | **Journal** | **Generic Drug/ BRAND NAME DRUG** | **Primary outcome** | **population** | **Study design** | **Result** |
| --- | --- | --- | --- | --- | --- | --- |
| Avramoff A  2007 | Int J Clin  Pharmacol Ther | Cyclosporin  NEORAL | AUC|_0 t_  AUC|_0 infinity_  C_max_ | 24  Healthy  Male | RCT  Cross-over  Single dose | Bioequivalence |
| Byakika-Kibwika  P 2008 | J Antimicrob Chemother | Triomune  ZERIT EPIVIR VIRAMUNE | AUC|_0 12h_  C_max_ | 18  Non Healthy  Male/female | RCT  Cross-over  Multiple dose | Bioequivalence  Not proven |
| Byakika-Tusiime J  2008 | PLoS ONE | Triomune  ZERIT EPIVIR VIRAMUNE | AUC|_0 12h_  C_max_ | 18  Non healthy  Male/female | RCT  Cross-over  Multiple dose | Bioequivalence  Not proven |
| Golden G  2008 | Clin Drug  Investig | Fazaclo  CLOZARIL | C_SSmax_  C_SSmin_  AUC _t_ | 36  Non healthy  Male/ female | RCT  Cross-over  Multiple dose | Bioequivalence |
| Di Girolamo G  2008 | Clin Ther | T4 Montpellier  SYNTHYROID | AUC|_0 t_  C_max_ | 24  Healthy  Male/female | RCT  Cross-over  Single dose | Bioequivalence |
| Hibberd AD  2006 | Transplantation | Cysporin  NEORAL | AUC|_0 12_  C_max_ | 32  Non healthy  Male/ female | RCT  Cross-over  Multi dose | Bioequivalence |
| Hosseinipour  2007 | AIDS | Triomune  EPIVIR ZERIT VIRAMUNE | AUC|_0 t_  C_0_  C_max_ | 12  Non healthy  Male/ female | RCT  Cross-over  Multiple dose | Bioequivalence non proved |
| Kees F  2006 | Ther Drug  Monit | Cicloral  NEORAL  SANDIMMUN | AUC|_0 infinity_  C_max_ | 12  Healthy  male | RCT  Cross-over  Single dose | Bioequivalence  Not proven |
| Kees F  2007 | Arch  Pharmacol | Neoimmun  NEORAL | AUC|_0 infinity_  C_max_ | 12  Healthy  Male / female | RCT  Cross-over  Single dose | Bioequivalence  Not proven |
| Lainesse A  2008 | Arzneimittel  forschung | Tacrolimus | AUC|_0 t_  AUC|_0 infinity_  C_max_ | 44  Healthy  Male/ female | RCT  Cross-over  Single dose | Bioequivalence |
| Marier JF  2006 | Int J Clin  Pharmacol Ther | Effavirenz  SUSTIVA | AUC|_0 t_  C_max_ | 42  Healthy  Male/ female | RCT  Cross-over Single dose | Bioequivalence |
| Masri MA  2007 | Transplant Proc | MMF  CELLCEPT | AUC|_0 t_  AUC|_0 infinity_  C_max_ | 24  Healthy  Male/ female | RCT  Cross-over Single dose | Bioequivalence |
| Park K  2007 | Clin Ther | Tacrobell  PROGRAF | AUC|_0 t_  AUC|_0 infinity_  C_max_ | 30  Healthy  Male/ female | RCT  Cross-over Single dose | Non inferiority |
| Pineyro-Lopez A  2005 | Clin Ther | Zaven  NEORAL | AUC|_0 t_  AUC|_0 infinity_  C_max_ | 36  Healthy  male | RCT  Cross-over Single dose | Bioequivalence |
| Tassaneeyakul W  2005 | J Pharm Pharm Sci | Cloril  CLOZARIL | AUC|_0 t_  C_max_ | 18  Non healthy  Male | RCT  Cross-over  Multiple dose | Bioequivalence |
| Tippabhotla SK  2008 | Int J Clin  Pharmacol Ther | Lopinavir ritonavir  KALETRA | AUC|_0 t_  AUC|_0 infinity_  C_max_ | 70  Healthy  Male/ female | RCT  Cross-over Single dose | Bioequivalence |
