# Supplementary material for: Quality of Reporting of Bioequivalence Trials Comparing Generic to Brand Name Drugs: A Methodological Systematic Review
Source: PLoS One. 2011 Aug 17;6(8):e23611. doi: 10.1371/journal.pone.0023611 (PMC3157430; doi:10.1371/journal.pone.0023611)
Supplement: Appendix S3 — Studies excluded because the name of the reference drug was not clearly reported. (DOC) [file pone.0023611.s007.doc]

Appendix S3: Studies excluded because the name of the reference drug was not clearly reported

1. Almeida A, Almeida S, Filipe A, Gagnon S, Mirapeix A, Girard B, et al. Bioequivalence study of two different coated tablet formulations of finasteride in healthy volunteers. Arzneimittelforschung. 2005;55(4):218-22.

2. Almeida S, Filipe A, Almeida A, Wong H, Caparros N, Tanguay M. Comparative bioavailability of two formulations of levofloxacin and effect of sex on bioequivalence analysis. Data from a randomised, 2 x 2 crossover trial in healthy volunteers. Arzneimittelforschung. 2005;55(7):414-9.

3. Almeida S, Portoles A, Terleira A, Filipe A, Cea E, Caturla MC. Comparative bioavailability/ bioequivalence of two different sertraline formulations: a randomised, 2-period x 2-sequence, crossover clinical trial in healthy volunteers. Arzneimittelforschung. 2005;55(4):191-7.

4. Chen J, Jiang WM, Xie YL, Jin L, Mei N, Liang XG. Evaluation of the bioequivalence and pharmacokinetics of two formulations of rizatriptan after single oral administration in healthy volunteers. Arzneimittelforschung. 2005;55(7):355-8.

5. Keller T, Cambon N, Genevray M, Crivelli F, Crivelli M, Dal BL, et al. Bioequivalence study of fluoxetine hydrochloride in healthy volunteers. Arzneimittelforschung. 2005;55(9):491-7.

6. Portoles A, Filipe A, Almeida S, Terleira A, Vallee F, Vargas E. Bioequivalence study of two different tablet formulations of carvedilol in healthy volunteers. Arzneimittelforschung. 2005;55(4):212-7.

7. Vlase L, Bodiu B, Leucuta SE. Pharmacokinetics and comparative bioavailability of two vinpocetine tablet formulations in healthy volunteers by using the metabolite apovincaminic acid as pharmacokinetic parameter. Arzneimittelforschung. 2005;55(11):664-8.

8. Yun MH, Kwon JT, Kwon KI. Pharmacokinetics and bioequivalence of haloperidol tablet by liquid chromatographic mass spectrometry with electrospray ionization. Arch Pharm Res. 2005 Apr;28(4):488-92.

9. Zhu X, Shin WG. Bioequivalence of diclofenac injection formulations assessed in Korean males. Int J Clin Pharmacol Ther. 2005 Nov;43(11):546-50.

10. Almeida S, Almeida A, Filipe A, Penedo C, Rocha A, Lainesse A, et al. In vitro disintegration and dissolution and in vivo bioequivalence of two alendronate once-weekly formulations. Arzneimittelforschung. 2006;56(2):84-9.

11. Almeida S, Filipe A, Almeida A, Antonijoan R, Garcia-Gea C, Gich I, et al. Comparative study on the bioequivalence of two different gabapentin formulations. A randomised, two-period, two-sequence, crossover clinical trial in healthy volunteers. Arzneimittelforschung. 2006;56(2):59-63.

12. Almeida S, Filipe A, Almeida A, Gich I, Antonijoan R, Puntes M, et al. Comparative study on the bioequivalence of two formulations of pravastatin. Data from a crossover, randomised, open-label bioequivalence study in healthy volunteers. Arzneimittelforschung. 2006;56(2):70-5.

13. Bienert A, Brzeziniski R, Szalek E, Dubai V, Grzeskowiak E, Dyderski S, et al. Bioequivalence study of two losartan formulations administered orally in healthy male volunteers. Arzneimittelforschung. 2006;56(11):723-8.

14. Gatchev E, Brater M, de Mey C. Bioequivalence of a novel oral metronidazole formulation. Arzneimittelforschung. 2006;56(8):612-6.

15. Gowda KV, Rajan DS, Mandal U, Selvan PS, Sam Solomon WD, Bose A, et al. Evaluation of bioequivalence of two formulations containing 100 milligrams of aceclofenac. Drug Dev Ind Pharm. 2006 Nov-Dec;32(10):1219-25.

16. Guilherme MC, Pereira DG, Galuppo MP, Mendes GD, Donato JL, De Nucci G. Bioequivalence of two lithium formulations in healthy volunteers. Arzneimittelforschung. 2006;56(7):524-8.

17. Ling G, Sun J, Xu X, Sun Y, He Z. Single and multiple dose bioequivalence evaluation of two brands of gliclazide modified release tablets in healthy Chinese male volunteers. Arzneimittelforschung. 2006;56(9):626-30.

18. Yusuf A, Al-Gaai E, Hammami MM. Bioequivalence evaluation of two rosiglitazone tablet formulations. Arzneimittelforschung. 2006;56(11):740-3.

19. Al Hawari S, AlGaai E, Yusuf A, Abdelgaleel A, Hammami MM. Bioequivalence study of two metformin formulations. Arzneimittelforschung. 2007;57(4):192-5.

20. Almeida S, Spinola AC, Filipe A, Trabelsi F, Farre A. Truncated AUCs in the assessment of the bioequivalence of topiramate, a long half-life drug. Arzneimittelforschung. 2007;57(5):249-53.

21. da Silva MA, Mendes FD, de Oliveira RA, Monif T, Patni A, Reyar S, et al. Comparative bioavailability study with two chlorpropamide tablet formulations in healthy volunteers. Arzneimittelforschung. 2007;57(9):591-8.

22. de Campos DR, Vieira NR, Bernasconi G, Barros FA, Meurer EC, Marchioretto MA, et al. Bioequivalence of two enteric coated formulations of pantoprazole in healthy volunteers under fasting and fed conditions. Arzneimittelforschung. 2007;57(6):309-14.

23. dos Santos Filho HO, Ilha JO, Silva LC, Borges A, Mendes GD, De Nucci G. Comparative bioavailability study with two amiodarone tablet formulations administered with and without food in healthy subjects. Arzneimittelforschung. 2007;57(9):582-90.

24. Gschwend MH, Erenmemisoglu A, Martin W, Tamur U, Kanzik I, Hincal AA. Pharmacokinetic and bioequivalence study of meloxicam tablets in healthy male subjects. Arzneimittelforschung. 2007;57(5):264-8.

25. Gschwend MH, Guserle R, Erenmemisoglu A, Martin W, Tamur U, Kanzik I, et al. Pharmacokinetics and bioequivalence study of ranitidine film tablets in healthy male subjects. Arzneimittelforschung. 2007;57(6):315-9.

26. Gschwend MH, Martin W, Erenmemisoglu A, Scherm M, Dilger C, Tamur U, et al. Pharmacokinetics and bioequivalence study of doxycycline capsules in healthy male subjects. Arzneimittelforschung. 2007;57(6):347-51.

27. Harahap Y, Prasaja B, Indriati E, Lusthom W, Lipin. Bioequivalence of ciprofloxacin tablet formulations assessed in Indonesian volunteers. Int J Clin Pharmacol Ther. 2007 Jun;45(6):373-6.

28. Hussein R, Lockyer M, Yusuf A, Al Gaai E, Abdelgaleel A, Hammami MM. Bioeqivalence assessment of two domperidone 1 tablet formulations. Arzneimittelforschung. 2007;57(5):269-73.

29. Hussein RF, Lockyer M, Hammami MM. Bioequivalence assessment of two capsule formulations of omeprazole in healthy volunteers. Arzneimittelforschung. 2007;57(2):101-5.

30. Jhee OH, Lee YS, Shaw LM, Jeon YC, Lee MH, Lee SH, et al. Pharmacokinetic and bioequivalence evaluation of two formulations of 100 mg trimebutine maleate (Recutin and Polybutin) in healthy male volunteers using the LC-MS/MS method. Clin Chim Acta. 2007 Jan;375(1-2):69-75.

31. Moreno RA, Boldrina L, Guermani A, Mazucheli J, Sverdloff C, Borges NC. Comparative bioavailability study of two phenoxymethylpenicillin potassium tablet formulations in healthy volunteers. Int J Clin Pharmacol Ther. 2007 Dec;45(12):669-76.

32. Sailer R, Arnold P, Erenmemisoglu A, Martin W, Tamur U, Kanzik I, et al. Bioequivalence study of sultamicillin suspensions. Arzneimittelforschung. 2007;57(4):232-7.

33. Sailer R, Arnold P, Erenmemisoglu A, Martin W, Tamur U, Kanzik I, et al. Comparative pharmacokinetics of two tablet formulations of amoxicillin: bioequivalence assessment. Arzneimittelforschung. 2007;57(4):227-31.

34. Setiawati E, Sukmayadi, Yunaidi DA, Handayani LR, Harinanto G, Santoso ID, et al. Comparative bioavailability cf two amlodipine formulation in healthy volunteers. Arzneimittelforschung. 2007;57(7):467-71.

35. Zhu de Q, Hu KL, Tao WX, Feng L, Duan H, Jiang XG, et al. Evaluation of the bioequivalence and pharmacokinetics of two formulations of secnidazole after single oral administration in healthy volunteers. Arzneimittelforschung. 2007;57(11):723-6.

36. Agarwal S, Gowda KV, Selvan PS, Chattaraj TK, Pal TK. Bioequivalence of two commercial preparations of escitalopram oxalate/clonazepam using a liquid chromatography-electrospray mass spectrometry method. Arzneimittelforschung. 2008;58(11):551-6.

37. Allegrini A, Nuzzo L, Scaringi AT, Felaco S, Pavone D, Toniato E, et al. Bioequivalence study of two capsule formulations of omeprazole in healthy volunteers. Arzneimittelforschung. 2008;58(8):385-8.

38. Bhaumik U, Ghosh A, Chakrabarty US, Mandal U, Bose A, Das A, et al. Evaluation of the bioequivalence of two faropenem formulations in healthy Indian subjects. Arzneimittelforschung. 2008;58(12):681-5.

39. Chakrabarty US, Mandal U, Bhaumik U, Chatterjee B, Ghosh A, Bose A, et al. Bioequivalence study of two capsule formulations containing diacerein 50 mg in healthy human subjects. Arzneimittelforschung. 2008;58(8):405-9.

40. Filipe A, Almeida S, Franco Spinola AC, Neves R, Trabelsi F, Torns A, et al. Bioequivalence study of two enteric-coated formulations of pantoprazole in healthy volunteers under fed conditions. Arzneimittelforschung. 2008;58(9):451-6.

41. Filipe A, Almeida S, Spinola AC, Trabelsi F, Ortuno J. Bioequivalence study of two letrozole tablet formulations. Single dose, randomized, open-label, two-way crossover bioequivalence study of letrozole 2.5 mg tablets in healthy volunteers under fasting conditions. Arzneimittelforschung. 2008;58(8):419-22.

42. Franco Spinola AC, Almeida S, Filipe A, Neves RI, Tanguay M, Yritia M. Bioequivalence of two formulations of levetiracetam. Int J Clin Pharmacol Ther. 2008 Nov;46(11):591-6.

43. Galan-Herrera JF, Poo JL, Maya-Barrios JA, de Lago A, Oliva I, Gonzalez-de la Parra M, et al. Bioavailability of two sublingual formulations of ketorolac tromethamine 30 mg: a randomized, open-label, single-dose, two-period crossover comparison in healthy Mexican adult volunteers. Clin Ther. 2008 Sep;30(9):1667-74.

44. Harahap Y, Sasongko L, Prasaja B, Indriati E, Lusthom W, Lipin. Comparative bioavailability of two estazolam tablet formulations in Indonesian healthy volunteers. Arzneimittelforschung. 2008;58(10):501-4.

45. Kano EK, Porta V, Koono EE, Schramm SG, Serra CH. Bioequivalence study of two oral formulations of cefadroxil in healthy volunteers. Arzneimittelforschung. 2008;58(1):42-7.

46. Koono EE, Kano EK, Schramm SG, dos Reis Serra CH, Porta V. Bioequivalence evaluation of two different tablet formulations of tinidazole in healthy volunteers. Arzneimittelforschung. 2008;58(11):598-601.

47. Mandal U, Das A, Agarwal S, Chakraborty U, Nandi U, Chattaraj TK, et al. Bioequivalence study of two formulations containing 400 mg dexibuprofen in healthy Indian subjects. Arzneimittelforschung. 2008;58(7):342-7.

48. Mendes FD, Patni AK, Reyer S, Monif T, Moreira LD, Ilha JO, et al. Comparative bioavailability study with two pantoprazole delayed-released tablet formulations administered with and without food in healthy subjects. Arzneimittelforschung. 2008;58(3):141-8.

49. Menon S, Kadam N, Patil G, Mhatre P. A randomized, crossover study to determine bioequivalence of two brands of dexibuprofen 400 mg tablets in healthy Asian adult male subjects of Indian origin. Int J Clin Pharmacol Ther. 2008 Jan;46(1):48-54.

50. Neves R, Almeida S, Filipe A, Spinola AC, Abolfathi Z, Yritia M, et al. Bioequivalence study of two different film-coated tablet formulations of losartan-hydrochlorothiazide in healthy volunteers. Arzneimittelforschung. 2008;58(8):369-75.

51. Pico JC, Dominguez G, Negri AL, Caubet JC, Terragno NA. Comparative pharmacokinetics of a single oral dose of two formulations of amlodipine. A randomized, single-blind, two-period, two-sequence, crossover study. Arzneimittelforschung. 2008;58(7):323-7.

52. Quetglas EG, Campanero MA, Sadaba B, Escolar M, Azanza JR. Bioequivalence of two oral formulations of triflusal capsules in healthy volunteers. Arzneimittelforschung. 2008;58(6):283-7.

53. Shin MC, Kim JK, Kim CK. Bioequivalence evaluation of two brands of lisinopril tablets by in vitro comparative dissolution test and in vivo bioequivalence test. Arzneimittelforschung. 2008;58(1):11-7.

54. Spinola AC, Almeida S, Filipe A, Tanguay M, Yritia M. Bioequivalence study of two tablet formulations of sildenafil. Arzneimittelforschung. 2008;58(3):122-5.

55. Zakeri-Milani P, Valizadeh H, Islambulchilar Z. Comparative bioavailability study of two cefixime formulations administered orally in healthy male volunteers. Arzneimittelforschung. 2008;58(2):97-100.
